# The Effect of the Distance from a Path on Abiotic Conditions and Vascular Plant Species in the Undergrowth of Urban Forests and Parks

**DOI:** 10.3390/ijerph19095621

**Published:** 2022-05-05

**Authors:** Kinga Kostrakiewicz-Gierałt, Katarzyna Gmyrek, Artur Pliszko

**Affiliations:** 1 Department of Tourism Geography and Ecology, Institute of Tourism, Faculty of Tourism and Recreation, University of Physical Education in Cracow, 31-571 Kraków, Poland; katarzyna.gmyrek@awf.krakow.pl; 2 Department of Taxonomy, Phytogeography and Palaeobotany, Institute of Botany, Faculty of Biology, Jagiellonian University, Gronostajowa 3, 30-387 Kraków, Poland; artur.pliszko@uj.edu.pl

**Keywords:** alien and native species, green spaces, trampling, urban soils, vegetation cover

## Abstract

Urban forests and parks are essential for the maintenance of biodiversity as well as human health and well-being. Residents and tourists commonly use urban forests and parks for recreational and sport purposes, contributing to changes in vegetation. This study aimed to assess the effect of distance from formal paths on the abiotic conditions, vegetation cover, as well as ecological diversity of vascular plant species in the undergrowth of urban forests and parks. The investigations were carried out in 2021 in 10 urban forests and 10 urban parks located in Kraków (southern Poland), using a total of 400 plots (1 × 1 m) situated in close (CL) and further (FU) vicinity of formal paths. We found a positive effect of the distance from the path on the depth of the compact soil layer, vegetation cover and height of the tallest shoot in the undergrowth of urban forests and parks. On the other hand, the distance from the path had a negative effect on the number of vascular plant species in the undergrowth in both forests and parks. Forests and parks differed significantly from each other in light intensity, the content of P in soil, depth of compact soil layer, number of species, as well as in cover-abundance of species representing different life forms, dispersal types, habitat affiliations and origins. Trampling leads to low plant cover and height of the undergrowth, as well as contributing to shallow localization of the compact soil layer near paths. Human movement on paths (walking, running, biking) with accompanying pets contributes to the successful dispersal of plants, resulting in high species richness. High light intensity in urban parks enhances the total number of species, cover-abundance of meadow and grassland plants, as well as cover-abundance of hemicryptophytes. The number of alien species was higher in parks than in forests, but the cover-abundance of alien plants was higher in forests than in parks. Urban forests are more suitable for the growth and biomass production of some alien herbs than urban parks, as mowing commonly used in parks appears to be an important factor in reducing their cover abundance. Regular fertilization and irrigation contribute to the high content of phosphorus in the soil, as well as to the high cover-abundance of meadow and grassland plants in urban parks. Urban forests enhance cover abundance of plants with dispersal mechanisms of the *Bidens* and *Lycopodium* types, whereas urban parks promote cover abundance of plants with the dispersal of the *Allium* type. Further study is needed to confirm the role of urban forests and parks in the preservation of ancient forest species, as well as to develop an appropriate design of paths that will allow the protection of vegetation and soil in urban forests and parks.

## 1. Introduction

Forests and parks in cities fulfill many important ecological, social and economic functions. They regulate microclimate, reduce surface runoff, produce oxygen, absorb air pollution, protect and purify soil and water, store and recycle organic matter, reduce noise and promote and preserve biodiversity [1,2,3,4,5,6,7,8]. They allow residents and tourists contact with nature, provide space for leisure, sport and recreation, improve well-being, promote physical and mental health and provide a space to establish contact or strengthen relationships with other people and pets [1,4,8,9,10,11]. They also provide space for education and small businesses and increase the land value [4].

Due to their easy access and attractiveness for residents and tourists, urban forests and parks are under constant human pressure [12,13]. The placement and use of various elements of infrastructure (e.g., fences, alleys, playgrounds, sports fields, outdoor gyms, rope courses, public toilets, gazebos, fountains, lanterns, benches, garbage or recycling bins, information boards, picnic and barbecue areas) in urban forests and parks contribute to the development of anthropogenic soils and ruderal plant communities [14,15]. Moreover, the degradation of soil and vegetation as a result of trampling and illegal garbage disposal is frequently observed in urban forests and parks [13,16]. This trampling can cause habitat fragmentation, reduce plant cover, change species composition, hamper woody plant regeneration and intensify soil erosion [13,14,17,18,19,20]. Illegal garbage disposal reduces the aesthetic value and can be a source of hazardous pollutants. Moreover, foraging through waste can harm or kill wild animals, and illegally dumped garbage may exacerbate wildfires [13,16,21]. It is also worth mentioning that soils in urban parks and forests are often contaminated by dog waste. According to Lee et al. [22], dog urine may have negative consequences for soil water-holding capacity and nutrient cycling in urban green infrastructure installations by directly decreasing the abundance and richness of soil microbial communities. Moreover, dog urine can cause nitrogen enrichment in the soil along the pathways [23].

Adequate management of urban forests and parks allows for the protection of the environment and biodiversity as well as the health and well-being of people [10,11,12,14,24]. Interestingly, old rural parks may serve as a refugium for forest species, and they can be more supportive of high biodiversity than remnants of wild forests [25]. Urban forests with well-preserved natural plant cover (wild urban forests) seem to be very different from typically designed city parks in their composition, structure and attractiveness for residents and tourists [9,26,27,28,29]. First of all, they are usually richer in native plant species than urban parks [28,29,30,31]. Secondly, they have a distinct and spontaneously layered structure with a dense tree layer, a well-developed shrub layer and typical forest undergrowth, whereas in many urban parks, the density of trees and shrubs is usually low, and the undergrowth resembles meadows, grasslands or lawns [26,31,32,33,34]. Alien vascular plants are commonly cultivated in urban green spaces due to their decorative values and resistance to unfavorable environmental conditions (e.g., drought, heat, air and soil pollution). However, many of them can escape from cultivation and establish themselves or even become invasive in cities and adjacent areas, posing a threat to native biodiversity and cultural heritage [35,36,37,38,39]. The progressing intensive development of cities causes significant changes in the functioning of forest and semi-natural ecosystems [40]. Studies involving the impact of anthropopressure on biodiversity in urban areas help to improve better planning and management of green spaces [14,27,30,41,42]. Formal paths are essential for proper movement in urban forests and parks [43,44]. Unfortunately, the frequent use of paths by residents and tourists may enhance the spread of invasive alien plants whose diaspores easily attach to shoes, clothes, sports equipment, vehicles or dog fur [18,45]. Moreover, in the close vicinity of paths, disturbances such as trampling, disposal of garbage or nitrogen enrichment by dog waste often occur [13,16,20,23]. In this study, we aimed to test the effect of the distance from formal paths on the abiotic conditions, vegetation cover, richness, ecological diversity and abundance of vascular plant species in the undergrowth of urban forests and parks.

## 2. Materials and Methods

### 2.1. Study Area

The study was conducted in Kraków, Lesser Poland Province, southern Poland, Central Europe, in 2021. Kraków is the second-largest city in Poland, with an area of 32,700 ha and 779,966 residents [46]. It is characterized by a temperate climate with an average annual air temperature of 9.3 °C and average annual precipitation of 730 mm [47]. Forests in Kraków cover a total area of 1377.34 ha, which is 4.21% of the city’s area. The largest share of the total forest area belongs to municipal forests managed by the Management of Urban Green Areas in Kraków (448.22 ha) and the municipal Wolski Forest managed by the Municipal Park and Zoological Garden Foundation in Kraków (397.41 ha).The forests managed by the State Forests cover an area of 270.82 ha, forests owned by natural persons cover 166.29 ha, State Treasury forests supervised by the Management of Urban Green Areas in Kraków an additional 59.10 ha and other owned forests amount to 35.5 ha. Forests in Kraków mainly perform protective, regulatory and social functions, resulting in their positive impact on the urban environment and the living conditions of the population. The majority of forests are deciduous forests of mesic habitats. The most valuable forests are Wolski Forest (the largest forest complex in the city with the nature reserves Panieńskie Skały and Bielańskie Skałki) and Mogilski Forest, with unique old oak and elm trees [48]. Currently, there are 50 public urban parks and one spa park in Kraków. They cover a total area of 462.1 ha, which is 1.41% of the city’s area.The largest public urban parks are the Polish Aviator’s Park (41.5 ha, without the Stanisław Lem Garden) and Błonia Krakowskie Park (41.2 ha). Most of the parks in Kraków have recreational, sports and tourist functions. In addition, 18 urban parks in Kraków have been registered as monuments protected by Polish law [48].

**Figure 1:**
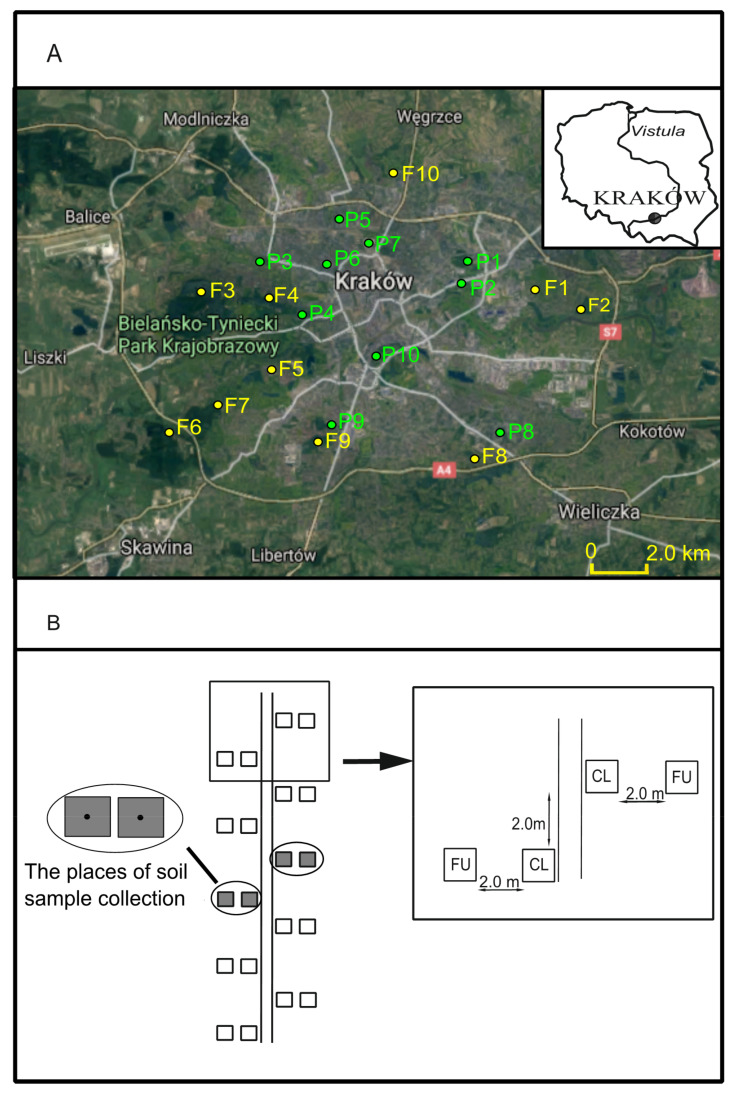
The location of study sites (**A**) and plot sampling design (**B**). The codes of study sites are explained in Table 1. CL indicates the plot located near the path, and FU indicates the plot located far from the path.

For the study, 10 urban forests with well-preserved natural and semi-natural forest vegetation and 10 urban parks with semi-natural and anthropogenic vegetation were selected (Figure 1A). The urban forests were represented by Łęgowski Forest, Mogilski Forest, Wolski Forest, the Forest at Sikornik Hill, the Forest at Pychowicka Hill, Tyniec Forest, Skotniki Forest, Rżącki Forest, Witkowice Forest and Borkowski Forest. Łęgowski Forest (20 ha) and Mogilski Forest (24 ha) are located in the eastern part of Kraków and include the remnants of the natural Ficario-Ulmetum minoris riparian forest. Wolski Forest (391.47 ha), Tyniec Forest (36 ha), the Forest at Sikornik Hill (24 ha), the Forest at Pychowicka Hill (17 ha) and Skotniki Forest (80.94 ha) are located in the western part of Kraków. They are mainly occupied by the remnants of a natural Tilio cordatae-Carpinetum betuli oak-hornbeam forest. Rżącki Forest (17 ha) is located in the southern part of Kraków and originated spontaneously on former farmland and is dominated by *Betula pendula* and *Populus tremula*. Borkowski Forest (70 ha) is located in the southern part of Kraków and includes the remnants of a natural oak-hornbeam forest (Tilio cordatae-Carpinetum betuli). Witkowice Forest (15 ha) is located in the northern part of Kraków and includes remnants of natural riparian (Ficario-Ulmetum minoris) and oak-hornbeam (Tilio cordatae-Carpinetum betuli) forests in the Bibiczanka River valley. The above-mentioned forests are mainly used for recreational activities by residents and tourists [49,50,51].

The urban parks were represented by the Polish Aviator’s Park, Dąbie Park, Decius Park, Twardowski Rocks Park, Stanisław Wyspiański Park, Henryk Jordan Park, Kleparski Park, Aleksandra Park, Solvay Park and Wojciech Bednarski Park. The Polish Aviator’s Park (43.6 ha) is located in the north-eastern part of Kraków. It is characterized by rich dendroflora and many sport attractions, such as a skatepark, pumptrack, street workout equipment, a multi-functional playground and a running route. Dąbie Park (9.16 ha) is located along the left bank of the Vistula River in the north-eastern part of the city. It includes recreation and sports infrastructure and is very suitable for observing wildlife. Decius Park (9.69 ha), one of the oldest parks in Kraków, is located in the north-western part of the city. It is considered to be a place for relaxation. Twardowski Rocks Park (34 ha) is located in the central part of Kraków. It is one of the most popular recreational areas in the city. It includes caves and former limestone quarries, as well as very valuable semi-natural thermophilic vegetation. Stanisław Wyspiański Park (2.57 ha) is located in the northern part of Kraków. The central part of the park is an open area with alleys, benches and a playground. Henryk Jordan Park (19.77 ha) is located in the northern part of Kraków. It is characterized by the presence of old trees typical of riparian forests (*Populus nigra* and *Ulmus* sp.). It includes many recreational and sports attractions, such as a basketball court, tennis court, playgrounds, fitness park, boule court, climbing wall, skate park, mini-street layout for young cyclists and a sledding hill. Kleparski Park (3.57 ha) is located in the northern part of Kraków. It surrounds Kleparz Fort, the only preserved fort in Kraków, and is frequently visited by residents and tourists for relaxation purposes. Aleksandra Park (5.20 ha) is located in the south-eastern part of Kraków, in the valley of the Bieżanowski stream. It includes valuable semi-natural habitats, such as dry sandy grasslands and wet meadows, and is used for sport and recreation. Solvay Park (8.79 ha) is located in the southern part of Kraków. It resembles a forest due to its rich dendroflora and is very suitable for bird watching. Wojciech Bednarski Park (8.24 ha) is located in the central-south part of Kraków. It is used for recreational and sports purposes [50,51].

### 2.2. Plot Sampling Design

Within each study site, one representative path was selected (Table 1). Then, along with each path, 10 pairs of 1 × 1 m plots were established. The pairs of plots were systematically distributed every 2 m (alternately on both sides of the path). Each pair consisted of a plot labeled CL (close), located 10 cm from the edge of the path, and a plot labeled FU (further), located 2 m from the CL plot. A total of 400 plots were recorded. The side of the path (left or right) where the plot sampling began was randomly selected by a coin toss. However, if any subsequent plot selected according to the sampling scheme was in a place occupied by a fallen tree or a trunk of a large tree, a new plot was established on the same side of the path, maintaining a 2 m-distance from the previous one. The location of study sites and plot sampling design are presented in Figure 1B.

### 2.3. Measurement of Abiotic Traits within the Plots

The field studies were conducted in summer, from 2 July 2021 to 19 July 2021. At one point in the central part of each plot, the light intensity at ground level, soil moisture, soil electrical conductivity and depth of the compacted soil layer were measured (with no repetitions). The light intensity was measured in sunny weather using a VOLTCRAFT LX-10 (0–199,900 lx) digital light meter. The soil moisture was measured before rainfall and when the plants in the undergrowth were dry using a handheld STELZNER 3000 device. The range of the moisture scale was from 1 to 10, where the values 1–3 indicated dry soils, 4–7 humid soils and 8–10 wet soils. The electrical soil conductivity was measured using a HANNA GROLINE direct soil conductivity tester. The depth of the compacted soil layer (understood as the depth at which the compacted soil layer began) was determined using an AGRETO penetrometer. Additionally, a total of 80 soil samples were collected from the central part of the CL and FU plots located in pairs 5 and 6 along the paths (Figure 1B). Each soil sample weighed approximately 0.5 kg and was collected from the top layer of soil, up to a depth of10 cm, using a stainless-steel soil spatula. In the laboratory, soil samples were dried at room temperature, then sieved (using a 2 mm sieve) and subjected to chemical analyses. The soil reaction, as well as the content of phosphorus (P), potassium (K), nitrate (N-NO_3_) and ammonium nitrogen (N-NH_4_) were determined using a VISOCOLOR^®^ kit (Macherey-Nagel, Düren, Germany), which assures the high-quality and accuracy of results.

### 2.4. Measurement of Vegetation Cover within the Plots

In each study plot, the vegetation cover traits were investigated in relation to vascular plant species occurring in the undergrowth (herb layer). The height of the tallest plant shoot was measured using a folding tape measure. The percentage of total vegetation cover was visually estimated with an accuracy of 5%. The vascular plant species were identified according to Csapodý [52], Muller [53] and Rutkowski [54]. The nomenclature followed Mirek et al. [55]. The cover abundance of each species was also visually estimated according to the Braun–Blanquet scale [56]. The explication of points on the scale is as follows:

“+”—species covers less than 1% of the plot area,
“1”—species covers 1–5% of the plot area,
“2”—species covers 6–25% of the plot area,
“3”—species covers 26–50% of the plot area,
“4”—species covers 51–75% of the plot area,
“5”—species covers 76–100% of the plot area,

For further calculations, the points of the Braun–Blanquet scale have been changed to the numerical values: 0.1, 1, 2, 3, 4, 5, respectively.

### 2.5. Selection of Ecological Traits of the Species

To assess the species’ response to human activities along the trails in urban forests and parks, we selected plant traits that were thought to be “ecologically meaningful” regarding persistence in stressful environments. These included life form, dispersal mode, habitat affiliation and species origin (native or alien). The list of species recorded in the plots is presented in Table A1. The life form (based on the Raunkiaer classification) was determined using the BiolFlor Database [57], LEDA traitbase [58] and Pladias Database [59]. The following life forms were included: phanerophytes (PH), chamaephytes (CH), hemicryptophytes (H), geophytes (G) and therophytes (T). In the case of the occurrence of more than one life form in one species, the most frequently mentioned life form in the cited databases was chosen. The dispersal mode was determined using the Pladias Database [59]. The following dispersal modes were included: *Allium* (mainly autochory, as well as anemochory, endozoochory, and epizoochory), *Bidens* (mainly autochory and epizoochory, as well as endozoochory), *Cornus* (autochory and endozoochory), *Epilobium* (mainly anemochory and autochory, as well as endozoochory and epizoochory), *Lycopodium* (mainly anemochory, as well as autochory, endozoochory, epizoochory and hydrochory), *Sparganium* (mainly autochory and hydrochory) and *Zea* (a dispersal strategy rarely or never dispersed by generative diaspores that do not form vegetative aboveground diasporas). A detailed description of the above-mentioned dispersal modes can be found in the paper by Sádlo et al. [60]. Habitat affiliation was assigned according to Matuszkiewicz [61], Zając and Zając [62] and Tokarska-Guzik et al. [63]. Habitat affiliation categories included (i) forest species (occurring in European mesotrophic and eutrophic deciduous forests from the class Querco-Fagetea Br.-Bl. et Vlieg., alder and shrub thickets from the class Alnetea glutinosae Br.-Bl. et R.Tx., coniferous forests from the class Vaccinio-Piceetea Br.-Bl. class), (ii) grassland species (occurring in calcareous grasslands from the class Festuco-Brometea Br.-Bl. et R.Tx., thermophilic fringe communities representing the classes Cratego-Prunetea Tx. and Trifolio-Geranietea sanguinei Th. Müller, sandy grasslands of the class Koelerio glaucae-Corynephoretea canescentis Klika in Klika et Novak, as well as *Nardus* grasslands and moors representing the class Nardo-Callunetea Prsg), (iii) meadow species (occurring in communities representing semi-natural and anthropogenic turf meadow communities from the class Molinio-Arrhenatheretea and alpine herbal and herbaceous plants from the class Betulo-Adenostyletea Br.-Bl.) and (iv) ruderal species (occurring in ruderal communities of perennial plants from the class Artemisietea vulgaris Lohm., Prsg et R. Tx. in R.Tx., natural and semi-natural nitrophilous communities from the subclass Galio-Urticenea (Pass.) Th. Müller in Oberd., moderately nitrophilous communities of summer therophytes from the class Bidentetea tripartite R.Tx., Lohm. et Prsg, nitrophilous communities of logging, trampled and ruderal areas from the class Epilobietea angustifolii R.Tx. et Prsg, semi-ruderal xerothermic pioneer communities from the class Agropyretea intermedio-repentis (Oberd. et al.) Müller et Görs, communities of arable fields and ruderal sites from the class Stellarietea mediae R.Tx., Lohm. et Prsg 1950, communities of small therophytes on moist and wet mineral substrates from the class Isoëto-Nanojuncetea Br.-Bl. et R.Tx., and communities of nitrophilic and halophilic plants from the class Cakiletea maritimae R.Tx. et Prsg). The origin of species was determined according to Tokarska-Guzik et al. [63] and Mirek et al. [55]. The invasive status of alien species followed Tokarska-Guzik et al. [63,64]. Taxa of uncertain geographical-historical status in the Polish flora [55] were excluded from the analysis of native and alien species. Moreover, plants identified only to genera, as well as taxa without data in a given category, were also excluded from the analyses.

### 2.6. Statistical Analyses

The mean light intensity, soil moisture, soil electrical conductivity, depth of the compacted soil layer, soil pH, content of P, K, N-NO_3_ and N-NH_4_ in the soil, percentage of total vegetation cover, number of species and height of the tallest plant shoot were calculated separately for CL and FU plots, as well as for forests and parks. The normal distribution of the untransformed data was tested using the Kołmogorov–Smirnov test, whereas the homogeneity of variance was verified using the Levene test at the significance level of *p* < 0.05. Two-way ANOVA analysis followed by the post-hoc Tukey test (in the occurrence of interaction) was performed to check the statistical significance of differences in (i) light intensity, (ii) soil moisture, (iii) soil electrical conductivity, (iv) depth of the compacted soil layer, (v) percentage of total vegetation cover, (vi) number of species and (vii) height of the tallest plant shoot, between (i) plots located at a different distance from tourist trails, and (ii) between plots located in forests and parks. The Mann–Whitney U test was applied to check the statistical significance of differences in the soil reaction and content of P, K, N-NO_3_ and N-NH_4_ between plots located (i) at a different distance from the paths and between (ii) plots located in forests and parks. The analyses were computed using STATISTICA software (version 13). The chi-square test was applied to check whether there were significant differences between the plots located in the forests and parks, as well as in plots located at different distances from the paths regarding the mean cover-abundance degree of species representing various life forms, dispersal modes, habitat affiliations and origins. The chi-square test was conducted using the interactive calculation tool [65].

## 3. Results

### 3.1. Light Intensity and Soil Conditions

**Figure 2:**
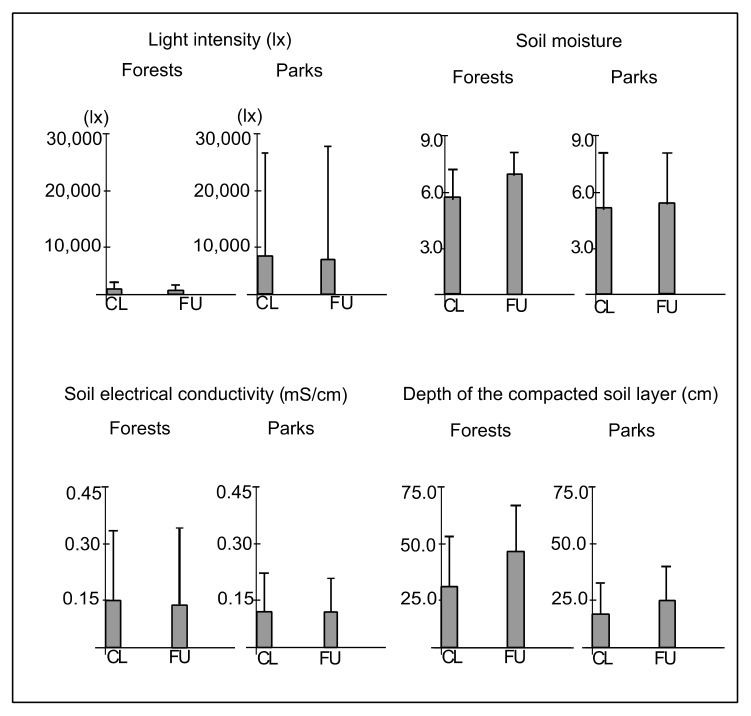
The mean (boxes) and standard deviation (whiskers) values of light intensity at ground level, soil moisture, soil electrical conductivity and depth of the compacted soil layer in closer (CL) and further (FU) plots located along paths in urban forests and parks.

**Figure 3:**
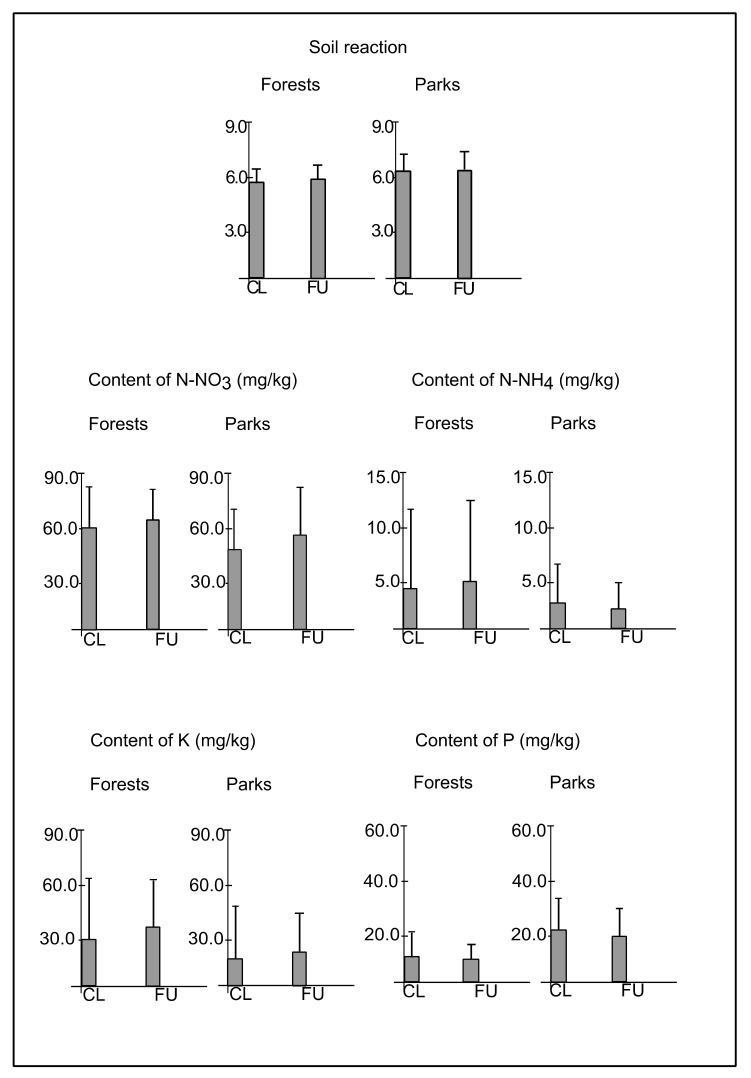
The mean (boxes) and standard deviation (whiskers) values of soil reaction, content of ammonium nitrogen (N-NH4), nitrate (N-NO_3_), potassium (K) and phosphorus (P) in soil samples of closer (CL) and further (FU) plots located along paths in forests and parks.

The mean light intensity in plots CL and FU in forests was 1262.1 (±1469.0) and 697.7 (±608.4) lx, respectively, whereas in parks, it was 8367.7 (±17,668.1) and 7967.9 (±18,428.4) lx, respectively. The differences between plots CL and FU in light intensity were statistically insignificant (F = 0.26; *p* = 0.61). However, the light intensity was significantly greater in parks than in forests (F = 29.95; *p* < 0.001) (Figure 2). The mean soil moisture in plots CL and FU in forests was 5.8 (±2.3) and 6.6 (±1.9), respectively, whereas in parks, it was 5.3 (±3.1) and 5.4 (±2.9), respectively. The differences between plots CL and FU in soil moisture were statistically insignificant (F = 1.75; *p* = 0.18). However, soil moisture was greater in forests than in parks (F = 23.13; *p* < 0.001) (Figure 2). The mean soil electrical conductivity in plots CL and FU in forests was 0.15 (±0.20) mS/cm and 0.14 (±0.21) mS/cm, respectively, whereas in parks, it was 0.12 (±0.10) mS/cm in both types of plots. The differences between plots CL and FU in soil electrical conductivity were statistically insignificant (F = 0.59; *p* = 0.44). Nevertheless, the soil conductivity was remarkably greater in forests than in parks (F = 24.60; *p* < 0.001) (Figure 2). The mean depth of the compacted soil layer in plots CL and FU in forests was 30.1 (±23.6) and 48.5 (±19.9) cm, respectively, whereas in parks, it was 16.9 (±12.7) and 24.8 (±15.9) cm, respectively. The depth of the compacted soil layer was significantly greater in plots FU than in CL (F = 39.98; *p* < 0.001), as well as in forests than in parks (F = 136.96; *p* < 0.001) (Figure 2). Moreover, ANOVA analysis confirmed the interactive effect of distance from path and type of study site on the depth of the compacted soil layer (F = 4.23; *p* ≤ 0.05), indicating the gradual decrease of the depth of compacted soil layer from plots FU in forests, through plots CL in forests and plots FU in parks, to plots CL in parks. The mean soil reaction in plots CL and FU in forests was the same and reached 5.9, while the standard deviation reached 0.78 and 0.93, whereas in parks, it was 6.2 (±0.52) and 6.4 (±0.55), respectively. The differences between plots CL and FU in forests (U = 200.0; *p* = 1.00) and parks (U = 174.0; *p* = 0.71), as well as between park and forest sites in plots CL (U = 169.0; *p* = 0.70) and FU (U = 144.0; *p* = 0.64) in soil reaction were statistically insignificant (Figure 3). The mean content of N-NO_3_ in plots CL and FU in forests was 60.4 (±31.6) and 65.0 (±32.6) mg/kg, respectively, whereas in parks, it was 48.3 (±29.3) and 56.9 (±29.7) mg/kg, respectively. The differences between plots CL and FU in forests (U = 184.5; *p* = 0.85) and parks (U = 166.0; *p* = 0.72), as well as between forests and parks in plots CL (U = 156.0; *p* = 1.57) and FU (U = 174.5; *p* = 0.77) in the content of N-NO_3_ were statistically insignificant (Figure 3). The mean content of N-NH_4_ in plots CL and FU in forests was 4.3 (±8.9) and 4.7 (±10.2) mg/kg, respectively, whereas in parks, it was 2.5 (±5.1) and 2.0 (±3.5) mg/kg, respectively. The differences between plots CL and FU in forests (U = 187.0; *p* = 0.71) and parks (U = 197.5; *p* = 0.88), as well as between forests and parks in plots CL (U = 180.0; *p* = 0.79) and FU (U = 192.5; *p* = 0.91) in the content of N-NH_4_ were statistically insignificant (Figure 3). The mean content of K in plots CL and FU in forests was 29.5 (±37.9) and 35.8 (±28.5) mg/kg, respectively, whereas in plots CL and FU in parks, it was 19.7 (±24.8) and 24.0 (±21.9) mg/kg, respectively. The differences between plots CL and FU in forests (U = 155.5; *p* = 0.62) and parks (U = 164.5; *p* = 0.67), as well as between forests and parks in plots CL (U = 177.0; *p* = 0.84) and FU (U = 155.5; *p* = 0.63) in the content of K were statistically insignificant (Figure 3). The mean content of P in plots CL and FU in forests was 9.3 (±8.6) and 8.5 (±6.3) mg/kg, respectively, whereas in parks, it was 22.5 (±14.5) and 20.5 (±15.1) mg/kg, respectively. The differences between plots CL and FU in forests (U = 194.5; *p* = 0.89) and parks (U = 175.0; *p* = 0.81) in the content of P were statistically insignificant. However, the content of P was significantly greater in parks than in forests in plots CL (U = 88.0; *p* < 0.01) and FU (U = 93.5; *p* < 0.01) (Figure 3).

### 3.2. Vegetation Cover Traits and Number of Species

**Figure 4:**
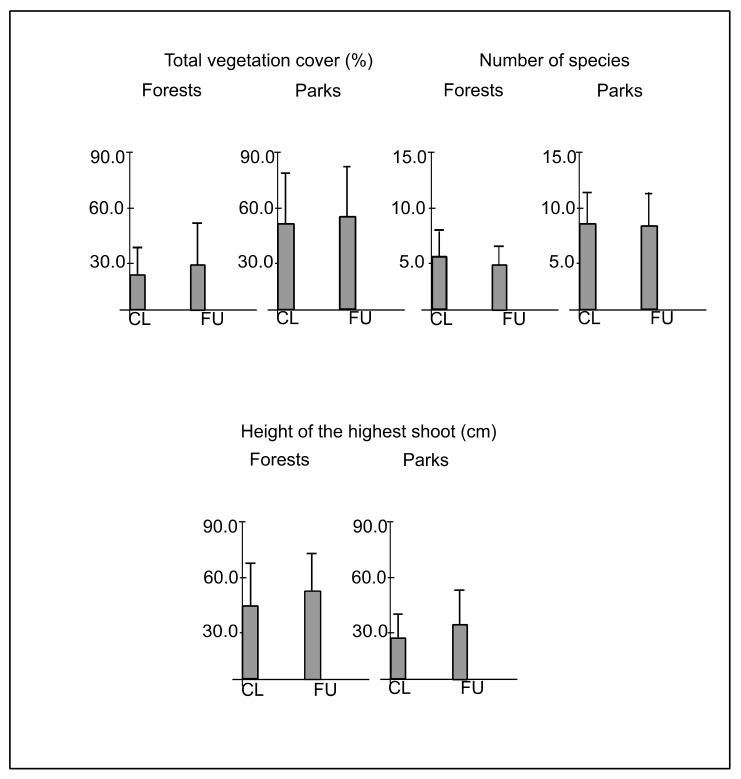
The mean (boxes) and standard deviation (whiskers) values of total vegetation cover, number of species and height of the tallest shoot in closer (CL) and further (FU) plots located along paths in forests and parks.

The mean vegetation cover in plots CL and FU in forests was 22.6 (±15.4)% and 28.0 (±23.2)%, respectively, whereas in parks, it was 53.4 (±26.6)% and 58.6 (±28.3)%, respectively. The vegetation cover was significantly greater in plots FU than CL (F = 5.49; *p* ≤ 0.05), as well as in parks than in forests (F = 149.00; *p* < 0.001) (Figure 4). The mean height of the tallest shoot in plots CL and FU in forests was 44.0 (±22.5) and 50.0 (±21.9) cm, respectively, whereas in parks, it was 27.4 (±14.5) and 33.2 (±18.5) cm, respectively. The height of the tallest shoot was significantly greater in plots FU than CL (F = 18.89; *p* < 0.001), as well as in forests than in parks (F = 61.41; *p* < 0.001) (Figure 4).

Altogether, 175 species of vascular plants were found in the plant cover, and some specimens were identified only to genera, namely *Carex, Crataegus, Dryopteris, Mentha, Rubus, Tilia* and *Viola* (Table A1). The total number of species in forests and parks was 102 and 127, respectively. Moreover, 48 species occurred only in forests, 73 species only in parks and 54 species were found both in forests and parks (Table A1). The mean number of species in plots CL and FU in forests was 5.9 (±2.0) and 4.6 (±1.9), respectively, whereas in parks, it was 8.9 (±3.3) and 8.8 (±3.4), respectively. The number of species was significantly greater in plots CL than FU (F = 5.22; *p* ≤ 0.05), as well as in parks than in forests (F = 184.65; *p* < 0.001) (Figure 4). ANOVA analysis confirmed the interactive effect of distance from path and type of study site on the number of species (F = 4.21; *p* ≤ 0.05), indicating the gradual decrease of the number of species from plots CL in parks through plots FU in parks and plots CL in forests, to plots FU in forests.

### 3.3. Ecological Characteristics of Species

**Figure 5:**
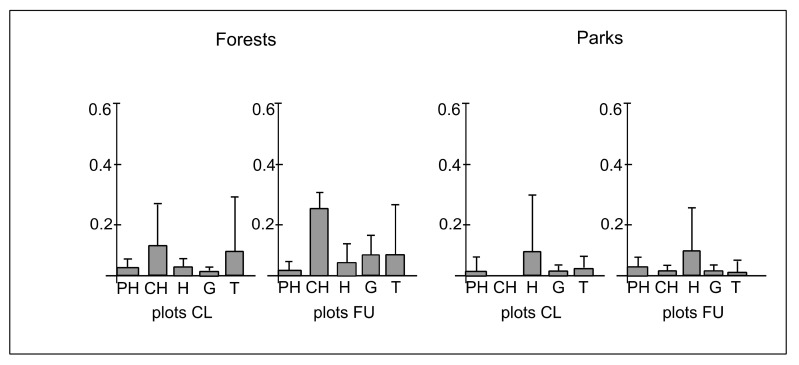
The mean (boxes) and standard deviation (whiskers) values of cover-abundance degree of species representing different life forms (PH—phanerophytes, CH—chamaephytes, H—hemicryptophytes, G—geophytes, T—therophytes) per plot in closer (CL) and further (FU) plots located along paths in forests and parks.

The life forms of the species were represented by phanerophytes, chamaephytes, hemicryptophytes, geophytes and therophytes (Table A1). The mean cover-abundance of phanerophytes in plots CL and FU in forests was 0.04 (±0.01), whereas in parks, it was 0.03 (±0.01). The mean cover-abundance of chamaephytes in plots CL and FU in forests was 0.11 (±0.22) and 0.24 (±0.12), respectively, whereas in parks, it was 0.00 and 0.01 (±0.01), respectively. The mean cover-abundance of hemicryptophytes in plots CL and FU in forests was 0.04 (±0.03) and 0.05 (±0.04), respectively, whereas in parks, it was 0.09 (±0.22) and 0.08 (±0.2), respectively. The mean cover-abundance of geophytes in plots CL and FU in forests was 0.02 (±0.02) and 0.07 (±0.04), respectively, whereas in parks, it was 0.01 (±0.01) and 0.02 (±0.01), respectively. The mean cover-abundance of therophytes in plots CL and FU in forests was 0.10 (±0.26) and 0.07 (±0.24), respectively, whereas in parks it was 0.02 (±0.03) and 0.01 (±0.03), respectively (Figure 5). The differences between plots CL and FU in forests (χ^2^ = 5.20; *p* = 0.26) and parks (χ^2^ = 1.36; *p* = 0.85) in cover-abundance of life forms were statistically insignificant (Figure 5). However, there were significant differences between forests and parks in plots CL (χ^2^ = 15.0; *p* < 0.01) and FU (χ^2^ = 17.32; *p* < 0.001). The chamaephytes and therophytes dominated in forests, whereas the hemicryptophytes dominated in parks (Figure 5).

**Figure 6:**
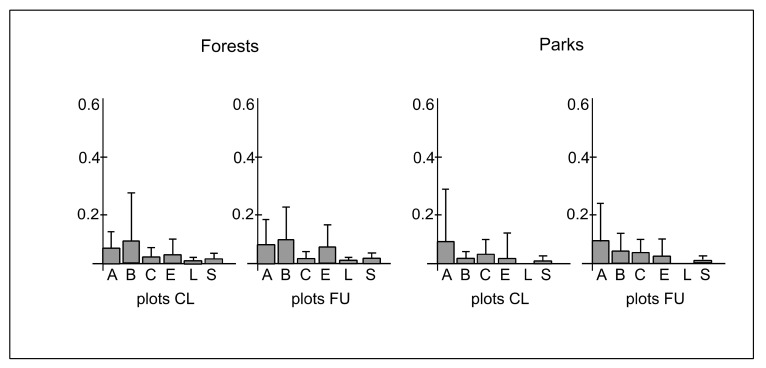
The mean (boxes) and standard deviation (whiskers) values of cover-abundance degree of species representing different dispersal types (A—*Allium*, B—*Bidens*, C—*Cornus*, E—*Epilobium*, L—*Lycopodium*, S—*Sparganium*) per plot in closer (CL) and further (FU) plots located along paths in forests and parks.

The dispersal types of the species were represented by *Allium, Bidens, Cornus, Epilobium, Lycopodium* and *Sparganium* (Table A1). The mean cover-abundance of species representing the *Allium* type in plots CL and FU in forests was 0.04 (±0.008) and 0.06 (±0.007), respectively, whereas in parks, it was 0.08 (±0.24) and 0.07 (±0.15), respectively. The mean cover-abundance of species representing the *Bidens* type in plots CL and FU in forests was 0.08 (±0.23) and 0.09 (±0.16), respectively, whereas in parks, it was 0.02 (±0.05) and 0.05 (±0.06), respectively. The mean cover-abundance of species representing the *Cornus* type in plots CL and FU in forests was 0.02 (±0.05) and 0.03 (±0.04), respectively, whereas in parks, it was 0.04 (±0.07) in both types of plots. The mean cover-abundance of species representing the *Epilobium* type in plots CL and FU in forests was 0.04 (±0.07) and 0.08 (±0.09), respectively, whereas in parks, it was 0.03 in both types of plots, while the standard deviation was 0.11 and 0.09, respectively. The mean cover-abundance of species representing the *Lycopodium* type in plots CL and FU in forests was the same and reached 0.02, whereas in parks this type of dispersal was absent. The mean cover-abundance of species representing the *Sparganium* type in plots CL and FU in forests was the same and reached 0.03 (±0.01), whereas in parks, it was also the same and reached 0.01(±0.01) (Figure 6). The differences between plots CL and FU in forests (χ^2^ = 2.07; *p* = 0.83) and parks (χ^2^ = 1.24; *p* = 0.74) in the cover-abundance of species representing different dispersal types were statistically insignificant (Figure 6). However, there were significant differences between forests and parks in plots CL (χ^2^ = 8.1; *p* ≤ 0.05) and FU (χ^2^ = 8.6; *p* ≤ 0.05). The *Allium* type dominated in parks, whereas the *Bidens* type dominated in forests (Figure 6).

**Figure 7:**
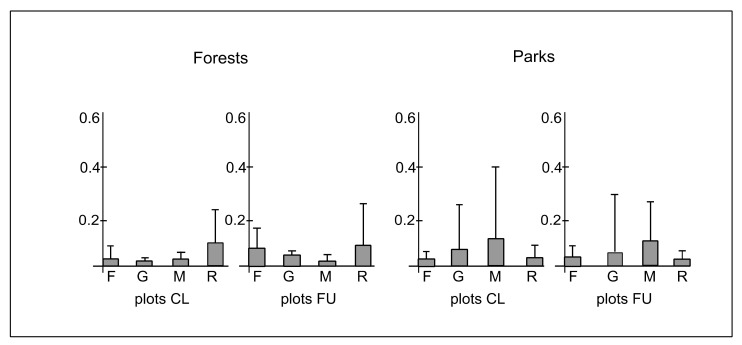
The mean (boxes) and standard deviation (whiskers) values of cover-abundance degree of species affiliated with different habitat types (F—forest, G—grassland, M—meadow, R—ruderal) per plot in closer (CL) and further (FU) plots located along paths in forests and parks.

The species affiliated with forest, grassland, meadow and ruderal habitats were found in plots CL and FU located in both forest and park sites (Table A1). The mean cover-abundance of species affiliated with forest habitats in plots CL and FU in forests was 0.03 (±0.03) and 0.05 (±0.06), respectively, whereas in parks, it was 0.03 (±0.03) and 0.04 (±0.05), respectively. The mean cover-abundance of species affiliated with grassland habitats in plots CL and FU in forests was 0.01 (±0.01) and 0.05 (±0.01), respectively, whereas in parks, it was 0.06 (±0.26) and 0.09 (±0.33), respectively. The mean cover-abundance of species affiliated with meadow habitats in plots CL and FU in forests was 0.02 (±0.01), whereas in parks, it was 0.12 (±0.31) and 0.10 (±0.22), respectively. The mean cover-abundance of species affiliated with ruderal habitats in plots CL and FU in forests was 0.08 (±0.18) and 0.09 (±0.22), respectively, whereas in parks, it was 0.03 in both types of plots, while the standard deviation reached 0.35 and 0.23, respectively (Figure 7). The differences between plots CL and FU in forests (χ^2^ = 0.90; *p* = 0.57) and parks (χ^2^ = 0.84; *p* = 0.93) in cover-abundance of species affiliated with different habitats were statistically insignificant. However, there were significant differences between forests and parks in plots CL (χ^2^ = 9.4; *p* ≤ 0.05) and FU (χ^2^ = 7.8; *p* ≤ 0.05). The species affiliated with ruderal habitats dominated in forests, whereas the species affiliated with grassland and meadow habitats dominated in parks (Figure 7).

**Figure 8:**
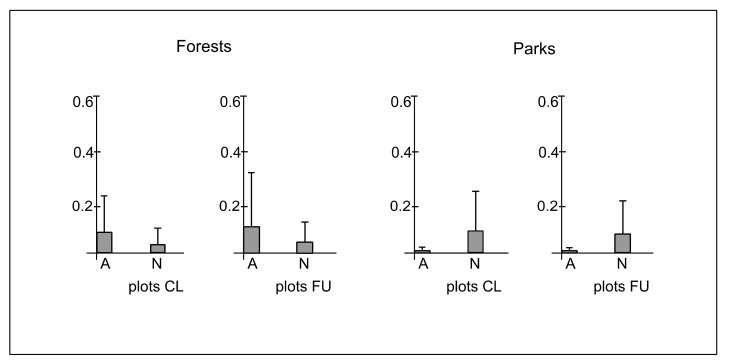
The mean (boxes) and standard deviation (whiskers) values of cover-abundance degree of alien (A) and native species (N) per plot in closer (CL) and further (FU) plots located along paths in forests and parks.

The total number of native and alien species was 140 and 30, respectively. There were also 5 species of uncertain status in the Polish flora. The number of alien species in forests and parks was 8 and 27, respectively. Moreover, among alien species, there were 15 species treated as invasive in Poland. The number of invasive alien species in forests and parks was 7 and 12, respectively (Table A1). The mean cover-abundance of native species in plots CL and FU in forests was 0.04 (±0.08) and 0.05 (±0.09), respectively, whereas in parks, it was 0.07, while the standard deviation reached 0.19 and 0.17, respectively, in both types of plots. The mean cover-abundance of alien species in plots CL and FU in forests was 0.07 (±0.18) and 0.11 (±0.23), respectively, whereas in parks, it was 0.01(±0.05) in both types of plots (Figure 8). The differences between plots CL and FU in forests (χ^2^ = 0.07; *p* = 0.78) and parks (χ^2^ = 0.27; *p* = 0.59) in cover-abundance of alien and native species were statistically insignificant. However, there were significant differences between forests and parks in plots CL (χ^2^ = 5.7; *p* ≤ 0.05) and FU (χ^2^ = 6.3; *p* ≤ 0.05). The cover-abundance of alien species was greater in forests, whereas the cover-abundance of native species was greater in parks (Figure 8).

## 4. Discussion

### 4.1. The Effect of Distance from the Path on Abiotic Conditions

The distance from the path did not affect the light intensity, soil moisture, soil electrical conductivity, soil reaction and content of N-NO_3_, N-NH_4_, K and P in the soil. However, it positively affected the depth of the compacted soil layer. The statistically significant differences between forests and parks were found only in the case of light intensity, depth of compacted soil layer and content of P in the soil. In the previous study conducted in Wolski Forest (based on the same plot sampling design), we also evidenced a lack of significant differences between plots CL and FU (located along the informal and formal tourist trails in forest interior and forest edge sites) in soil moisture, soil reaction and content of N-NO_3_, N-NH_4_, K and P in the soil [20]. Most likely, the distance from the path was too short to find significant differences between plots CL and FU in these parameters. On the other hand, in the previous study [20], we demonstrated that light intensity can be greater in plots CL than in FU in the case of informal and formal trails located in forest interior sites, as well as in the case of informal trails located in forest edge sites. Nevertheless, the homogeneity of light conditions along formal paths was observed in other temperate forests [66], as well as in forest edge sites [20]. Generally, in forests and parks, the light intensity in the undergrowth (herb layer) depends on the cover-abundance of woody plants occurring in the tree and shrub layers. The density of trees and shrubs is usually lower in parks than in forests, allowing the development of many light-demanding meadow and grassland plant species [26,31,32,33,34]. In urban parks, unlike wild urban forests, many plants are artificially distributed in accordance with the planting design and regularly cared for by greenery and public sanitation workers [26,27]. The planting of trees and shrubs at large distances from each other and pruning, as well as regular mowing (which hamper the spontaneous regeneration of woody plants), increase the light intensity in the undergrowth. Our study confirmed the pattern that light intensity in the undergrowth is greater in parks than in forests. The dense canopy of trees and shrubs, as well as the dense and thick litter layer, provides and preserves the high soil moisture in many deciduous forests [67]. Therefore, the low density of trees and shrubs, as well as commonly practiced leaf raking and litter removal in parks, may negatively affect soil moisture. On the other hand, the dense turf of herbaceous plants in parks may increase soil moisture. The fact that vegetation cover in the undergrowth was greater in parks than in forests may explain the lack of differences in soil moisture, although the light intensity was lower in forests.

The soil electric conductivity can be affected by various factors such as soil texture, temperature, moisture level, irrigation, amount of fertilizers and salinity [68]. The effect of distance from the road on soil electric conductivity has been tested along the road for motor traffic in Kraków by Pająk et al. [69]. As a result of the chemical de-icing of the road with salt, the authors evidenced higher soil electrical conductivity at a distance of 1 m than at a distance of 2 m from the road. Moreover, the values of soil electric conductivity in their study were the highest in March. In our study, the distance from the path did not affect soil electric conductivity. However, the values of soil electric conductivity in forests and parks in Kraków were similar to those evidenced by Pająk et al. [69] (for samples collected in July). To the best of our knowledge, salt has not been used on the paths in study sites in the winter of 2021. According to Shannon et al. [70], forests show remarkably lower soil electrical conductivity than urban parks as the effect of road salt application. Nevertheless, we found that soil electrical conductivity was greater in forests than in parks. It is difficult to explain unequivocally what factors caused such a result. In addition to the previously mentioned factors, the increase in soil electrical conductivity may be caused by illegal dumping [71,72,73]. According to The Management of Urban Green Areas in Kraków [51], tens of tons of rubbish are collected annually from municipal forests. It is also worth mentioning that the application of salt in winter increases soil pH near the roads [69]. Moreover, the alkalization of soils near the roads may be caused by asphalt, which is often used to cover the surface of the soil in urban paths, sidewalks and roads [74]. Generally, the soil pH in forests and parks was slightly acidic and lower than evidenced in other studies conducted in Kraków [20,69].

The heterogeneity of urban soils is mainly referable to different land uses. The nutrient content in urban soils increases due to fertilization and pollution, and the highest levels of soil nutrients can be found on roadsides and residential areas [75]. The content of phosphorus in the soil was significantly greater in parks than in forests. This result can be explained by management practices in urban parks, such as regular fertilization and irrigation [76,77]. Additionally, dog waste can be a source of phosphorus in the soils of urban parks. Paradeis et al. [78] noticed such a phenomenon in off-leash dog parks, while Bonner and Agnew [79] noticed the high phosphorus levels in the soil of urban recreation areas maintained three years after dogs had been banned. The soils near the paths and roads are particularly prone to compaction due to trampling and road traffic. Soil compaction is a physical form of soil degradation that affects soil structure, limits water and air infiltration and reduces root penetration in the soil [80]. On the other hand, soil compaction is crucial for the construction process of many elements of infrastructure, such as roads, pavements and squares [81]. Trampling is commonly observed in urban areas and may lead not only to soil compaction but also to the reduction in plant cover, changes in species composition, habitat fragmentation, as well as soil erosion [13,14,18,19,20]. The compact soil was situated shallower in the plots CL than in FU, suggesting the negative effect of the construction of the paths and trampling on soil structure. Moreover, our results indicated that the soils along the paths are less compacted in urban forests than in urban parks, with a gradual decrease of the depth of compacted soil layer from plots FU in forests, through plots CL in forests and plots FU in parks, to plots CL in parks.

### 4.2. The Effect of Distance from the Path on Vegetation Cover and Number of Species

The distance from the path positively affected both vegetation cover and the height of the tallest shoot in the undergrowth. This result can be explained by trampling, as evidenced in other studies [19,20,82]. However, the total plant cover is not always greater in plots located further from the paths, suggesting the influence of other environmental factors, such as vegetation type, light intensity and width of the path [20]. The great cover-abundance of the undergrowth in urban parks may be explained by the high share of meadow and grassland plant species, which often form dense clusters of shoots, such as *Arrhenatherum elatius* [83], *Holcus lanatus* [84], *Lolium perenne* [85] and *Thymus serphyllum* [86], as well as by regular treatment with fertilizers and irrigation that enhance plant biomass [76,77]. Moreover, the greater height of the undergrowth in forests may be a result of lower light intensity [87].

The distance from the path had a negative effect on the number of vascular plant species in the undergrowth. We observed the same pattern along formal tourist trails in forest interior and forest edge sites in Wolski Forest [20]. The paths in urban areas enhance plant migration by supporting the seed dispersal through passing humans and animals or attachment to vehicles and equipment; therefore, the number of plant species can be greater close to the paths [20,45]. Moreover, we evidenced that the number of species was greater in parks than in forests, with a gradual decrease from plots CL in parks, through plots FU in parks and plots CL in forests, to plots FU in forests. Most likely, this result can be explained by differences in light intensity. In edge forest sites in Wolski Forest, we found more plant species than in interior forest sites, which differed significantly in light intensity [20]. Similarly, Moszkowicz et al. [88] evidenced that the great light intensity due to low tree cover enhances species richness in parks in Kraków. Furthermore, the species richness may be positively impacted by the low intensity of mowing. According to Sehrt et al. [89], the richness of plant species in urban grasslands increases with reduced mowing intensity. Nevertheless, many parks in Kraków are intensively mowed [88]. We also evidenced that the number of species increased with decreasing depth of the compacted soil layer from plots FU in forests, through plots CL in forests and plots FU in parks, to plots CL in parks. The great soil compactness in urban areas is commonly caused by the construction of buildings and various elements of infrastructure, as well as by car traffic and trampling [80,81,90]. Many grassland, meadow and ruderal plant species are well adapted to trampling and frequently occur on roadsides, i.e., *Juncus tenuis, Lolium perenne, Poa annua, Plantago major* and *Trifolium repens* [61,91]. Perhaps the depth of the compacted soil layer was not so shallow as to significantly reduce the species richness. In addition, the richness of herbaceous plants in urban parks in Kraków depends on many different factors such as the size of the area, topography (height differences), presence of a migration corridor and presence of natural elements [88].

### 4.3. The Effect of Distance from the Path on Ecological Diversity and Abundance of Species

The distance from the path did not affect the cover-abundance of species representing different life forms, habitat affiliation, dispersal mode and origin. Nevertheless, the ecological diversity and abundance of species differed between forests and parks. Interestingly, in our previous study [20], we demonstrated the differences between plots CL and FU in the cover-abundance of species representing various life forms and dispersal modes, in the cases of forest interior and forest edge sites, along informal and formal tourist trails. We also evidenced the differences between plots CL and FU in the cover-abundance of alien and native species in the case of forest edge sites, along informal and formal trails, as well as the differences in the cover-abundance of species representing various habitat affiliations in the case of formal trails in forest interior sites and informal and formal trails in forest edge sites [20]. In this study, we showed the dominance of hemicryptophytes in parks and the dominance of chamaephytes and therophytes in forests. Urban parks, unlike forests, often comprise large patches of meadow and grassland vegetation that are rich in hemicryptophytes [92,93]. Moreover, meadow plants representing hemicryptophytes are commonly planted in urban parks, including the area of Kraków [94]. We recorded many native hemicryptophytes, such as *Achillea millefolium, Avenula pubescens, Bellis perennis, Festuca arundinacea, Holcus lanatus, Lolium perenne, Potentilla anserina, Taraxacum officinale, Trifolium pretense* and *Trifolium repens,* that are commonly distributed in parks of European cities [93]. The considerable cover-abundance degree of chamaephytes in forests might be a result of successful vegetative propagation of *Galeobdolon luteum* and *Stellaria holostea* due to their guerrilla growth strategy [95], leading to quick spreading and finding favorable microsites within a heterogenous area. The occurrence of populations of *Stellaria holostea* in urban forests due to prolonged clonal growth and generative propagation assuring genetic variability was evidenced by Wódkiewicz and Gruszczyńska [96]. Moreover, the considerable abundance of therophytes in recreationally used urban and suburban forests was observed by Vakhlamova et al. [97]. The aforementioned authors argued that the occurrence of therophytes, as well as alien taxa, is an effect of human-mediated disturbances such as trampling and damage of ground and vegetation occurring, among others, by walking, biking or playing sports.

As is well known, urbanization favors the influx of alien plant species. The richness, diversity and distribution of alien plant species in urban areas depend on various environmental factors and human activities. In artificial habitats, the highest species richness is found in sites with relatively high levels of urbanization, while in semi-natural habitats, the highest species richness occurs in the less urbanized sites. Moreover, in semi-natural habitats, most of the richness of alien and native species is associated with the distance to the city center, and a high level of urbanization is associated with a large abundance of alien species in both artificial and in semi-natural habitats in riparian areas [98]. According to Duchesneau et al. [99], the richness of alien species in urban forest fragments is primarily affected by residential layout, recent construction events and nearby roads. Moreover, Vojík et al. [38] evidenced that the distribution of alien taxa in parks is affected by altitude, % of the area with semi-natural vegetation and % of the area with English landscape (a nature-like part of the park with much less intense regular management). The most important factors for invasive alien species distribution in urban areas are river and alluvial soils, forests and related rusty soils and places of intensive human activities, including areas of urbisols and industriosols [100]. In our study, the number of native species was greater than the number of alien species both in plots CL and FU, as well as in forests and parks, but more alien species were found in parks than in forests. The dominance of native plants in urban areas has been repeatedly reported by many authors (i.e., [29,30,31,35]). Interestingly, urban parks are viewed as sources of alien plant species escaping from cultivation, but they can also serve as habitats for threatened native plants [38]. In our study, we observed that some alien plants cultivated in parks spread along paths in parks, i.e., *Acer saccharinum, Quercus rubra* and *Robinia pseudoacacia*, but we did not find any rare, threatened or protected native species. Moreover, we found archaeophytes (i.e., *Bromus sterilis*, *Capsella bursa-pastoris*, *Euphorbia peplus*, *Fallopia *convolvulus*, *Geranium pussilum*, *Lactuca serriola*, *Lamium album*, *Lamium purpureum*, *Melandrium album*, Myosotis arvensis, Setaria viridis*, *Veronica arvensis* and *Vicia tetrasperma*) only in parks. According to Moszkowicz et al. [88], the share of archaeophytes increases in the herb layer of isolated and flat urban parks in Kraków. As in other cities in Poland [100,101], urban areas in Kraków are under invasion by many neophytes. We found four invasive neophytes that occurred both in forests and parks, namely *Impatiens parviflora*, *Padus serotina*, *Quercus rubra* and *Solidago gigantea*. Although the number of alien species was much lower in forests than in parks, the cover-abundance of alien species was higher in forests. This suggests that urban forests are more suitable for the growth and biomass production of some alien herbs than urban parks. It was particularly visible in the case of *Impatiens parviflora*, one of the most common invasive alien plants in Kraków, which achieved higher cover-abundance in forests than in parks. Most likely, mowing, which is commonly practiced in urban parks in Kraków [88], effectively inhibits the abundance of invasive alien species in the undergrowth. Nevertheless, the observed presence of invasive alien plants in urban parks is also worrying, especially in the case of ruderal species producing numerous small-sized seeds that can be dispersed over long distances by wind, such as *Conyza canadensis* [102] and *Solidago canadensis* [103]. Considering that invasive alien plants can be found in various urban habitats in Kraków, their control is needed.

In urban areas, plant dispersal is particularly impacted by human activities. Many native and alien plants use paths, roads and railway tracks as corridors for successful dispersal, as their diaspores can be easily transported on vehicles and human clothing [45,104,105]. On the other hand, urbanization may negatively affect the dispersal of zoochorous plants, as many wild animals avoid urban areas or restrict their movements within urban habitats [106].In this study, we showed that the species of the *Allium* dispersal type (mainly autochory, as well as anemochory, endozoochory and epizoochory) had the greatest cover-abundance in parks, whereas the species of the *Bidens* type (mainly autochory and epizoochory, as well as endozoochory) had the greatest cover-abundance in forests. According to Sádlo et al. [60], the *Allium* type dominates in both anthropogenic vegetation and forests. Similarly, we observed the dominance of the *Allium* type in interior forest sites in Wolski Forest, especially along informal tourist trails [20]. The low cover-abundance of the *Bidens* type in parks corresponds to the findings by Moszkowicz et al. [88], who evidenced that the number of species spreading via animals increases in sites situated in less urbanized environments of Kraków. Moreover, the occurrence of the *Lycopodium* dispersal type (mainly anemochory, as well as autochory, endozoochory, epizoochory and hydrochory) only in forests seems to support the findings of Sádlo et al. [60], who evidenced that this dispersal type has a greater share in woodlands than in anthropogenic vegetation. The rapid development of cities in recent decades has significantly contributed to the decline of forest ecosystems and the development of anthropogenic habitats in urban areas. Apart from significant soil transformations, changes in water conditions or air pollution, the increasing fragmentation of natural and semi-natural habitats and their isolation from each other in large cities adversely affect the maintenance of many native species [40]. On the other hand, highly urbanized and industrialized areas can also have a positive influence on vegetation and the ecological diversity of species. For example, urban parks are potential places for the growth of various types of vegetation and also for increasing biodiversity [92]. In our study, considering the cover-abundance of species, ruderal plants dominated in forests, whereas meadow and grassland plants dominated in parks. This result can be explained by differences in light conditions, as numerous light-demanding meadow and grassland plants commonly grow in urban parks [92], as well as on the edges of urban forests [20]. Moreover, many meadow and grassland plants occurring in urban parks are planted and supported by urban greenery workers who use fertilization and irrigation to enhance plant condition and abundance [76]. However, in some urban areas, a high level of urbanization is associated with a large abundance of ruderal species in both artificial and semi-natural habitats [98]. Although the cover-abundance of forest species was similar between forests and parks, the number of forest species was greater in forests than in parks (Table A1). Interestingly, in urban forests, we found many species typical of ancient forests (forests with continuous habitat history and no record of agricultural use), such as *Actaea spicata*, *Ajuga reptans*, *Anemone nemorosa*, *Asarum europaeum*, *Carex remota*, *Carex sylvatica*, *Circaea lutetiana*, *Convallaria majalis*, *Galeobdolon luteum*, *Galium odoratum*, *Lathyrus vernus*, *Melica nutans*, *Mercurialis perennis*, *Millium effusum*, *Moehringia trinervia*, *Paris quadrifolia*, *Poa nemoralis*, *Polygonatum multiflorum*, *Pteridium aquilinum*, *Pulmonaria obscura*, *Symphytum tuberosum* and *Viola reichenbachiana* [107]. This suggests that urban forests can preserve elements of natural forest vegetation. Furthermore, in urban parks, ancient forest species also occurred (i.e., *Anemone nemorosa, Epilobium montanum* and *Viola reichenbachiana*). However, some ancient forest species (geophytes) seem to be underestimated since our study was conducted in summer.

## 5. Conclusions

Green areas in cities are essential for the maintenance of biodiversity as well as human health and well-being. Residents and tourists commonly use urban forests and parks for recreational and sports purposes. The richness, abundance and ecological diversity of vascular plants in urban areas depend on various environmental factors and human activities. We showed a positive influence of the distance from the path on the depth of compact soil layer, vegetation cover and height of the tallest shoot in the undergrowth of urban forests and parks. On the other hand, the distance from the path had a negative effect on the number of vascular plant species in the undergrowth in both forests and parks. The trampling and other mechanical damage to vegetation occurring near paths contribute to low cover and height of undergrowth and shallow localization of the compact soil layer. Human movement on paths (walking, running, biking) with accompanying pets contributes to the successful dispersal of plants resulting in a high number of species (in general), as well as alien species near paths. The soil in urban parks had greater compactness than in forests suggesting greater degradation by intensive trampling, as well as by construction of paths with artificial elements. Light intensity in the undergrowth was higher in urban parks than in urban forests due to the low cover-abundance of trees and shrubs. The high light intensity in urban parks enhances the total number of species, cover-abundance of meadow and grassland plants, as well as cover-abundance of hemicryptophytes. The similarity between urban forests and parks in floristic composition was low, sharing only 30% of plant species recorded. The number of alien species was higher in parks than in forests, but the cover-abundance of alien plants was higher in forests than in parks. Urban forests are more suitable for the growth and biomass production of some alien herbs (*Impatiens parviflora*) than urban parks, as mowing is commonly used in parks and appears to be an important factor in reducing their cover-abundance. On the other hand, regular fertilization and irrigation contribute to the high content of phosphorus in the soil, as well as the cover-abundance of meadow and grassland plants in urban parks. Urban forests enhance cover-abundance of plants dispersing by the *Bidens* and *Lycopodium* types, whereas urban parks promote cover-abundance of plants dispersing by the *Allium* type. Urban forests can preserve remnants of natural forest vegetation by having a high number of ancient forest species in the undergrowth. However, the importance of urban forests and parks in the preservation of ancient forest species needs further study, including geophytes occurring in spring. The management of green areas in cities should be a kind of compromise between the needs of people and wildlife. Unfortunately, the desire to make forests and parks more accessible to people may intensify soil degradation and fragmentation of plant cover, as well as enhance the process of invasion of alien plant species. To better protect native diversity in urban forests and parks, more detailed studies should be undertaken in the future, taking into account factors such as the number of visitors, frequency of visits and number and width of paths. Finally, it is worth emphasizing that urban forests and parks are excellent places to observe anthropogenic changes in vegetation, which can be used in environmental education. On the other hand, the spread of invasive alien species in urban areas should be monitored and controlled.

## Figures and Tables

**Table 1:** Characteristics of study sites.

Name of Study Site	Code	GPS Coordinates	Altitude (m a.s.l.)	Width of the Path (cm)	Surface of the Path
Łęgowski Forest	F1	N50°03.390′ E20°01.814′	203	220	Natural
Mogilski Forest	F2	N50°03.233′ E20°03.341′	210	260	Anthropogenic (asphalt)
Wolski Forest	F3	N50°03.327′ E19°51.468′	331	190	Natural
Forest in Sikornik Hill	F4	N50°03.509′ E19°53.236′	258	130	Natural
Forest in Górka Pychowicka	F5	N50°01.903′ E19°52.977′	240	230	Natural
Tyniec Forest	F6	N50°00.633′ E19°49.712′	277	250	Natural
Forest in Skotniki	F7	N50°01.251′ E19°51.120′	209	220	Anthropogenic (gravel)
RżąckiForest	F8	N50°00.342′ E19°59.797′	266	100	Natural
Borkowski Forest	F9	N50°00.608′ E19°54.795′	260	300	Anthropogenic (gravel)
Witkowice Forest	F10	N50°06.471′ E19°57.001′	249	100	Natural
PolishAviator’s Park	P1	N50°04.377′ E19°59.441′	223	160	Anthropogenic (asphalt)
Dąbie Park	P2	N50°03.608′ E19°59.055′	206	220	Natural
Decius Park	P3	N50°03.855′ E19°52.384′	219	260	Anthropogenic (asphalt)
Twardowski Rocks Park	P4	N50°02.366′ E19°54.154′	220	300	Anthropogenic (asphalt)
StanisławWyspiański’sPark	P5	N50°05.144′ E19°55.245′	235	310	Anthropogenic (asphalt)
Henryk Jordan’s Park	P6	N50°03.864′ E19°55.087′	206	230	Anthropogenic (asphalt)
Kleparski Park	P7	N50°04.572′ E19°56.310′	226	310	Natural
Aleksandra’s Park	P8	N50°00.827′ E20°00.828′	243	160	Anthropogenic (gravel)
Solvay Park	P9	N50°00.905′ E19°55.591′	273	180	Natural
Wojciech Bednarski’s Park	P10	N50°02.548′ E19°57.000′	218	370	Anthropogenic (asphalt)

## Data Availability

Not applicable.

## Appendix A

**Table A1:** Vascular plants recorded within the close (CL) and further (FU) plots in urban forests and parks in Kraków and their ecological characteristics. Abbreviations: 1—presence of the taxon in the study plot; 0—absence of the taxon in the study plot; PH—phanerophyte, CH—chamaephyte, H—hemicryptophyte, G—geophyte, T—therophyte; nd—not determined (no data); ?—taxon of uncertain status in the Polish flora, likely to be an anthropophyte; *—invasive species in Poland.

Taxon (Speciesorgenus)	Presence or Absencein Plots				Life Form	Dispersalmode	Habitat Affiliation	Origin
	Forests		Parks					
	CL	FU	CL	FU				
*Acer platanoides* L.	1	1	1	1	PH	*Epilobium*	Forest	Native
*Acer pseudoplatanus* L.	1	1	1	1	PH	*Epilobium*	Forest	Native
*Acer saccharinum* L.	0	0	0	1	PH	*Epilobium*	Ruderal	Alien
*Achillea millefolium* L.	0	0	1	1	H	*Allium*	Meadow	Native
*Actaea spicata* L.	1	0	0	0	H	*Cornus*	Forest	Native
*Aegopodium podagraria* L.	1	1	1	1	H	*Allium*	Forest	Native
*Agrimonia eupatoria* L.	1	0	1	1	H	*Bidens*	Grassland	Native
*Agrostis capillaris* L.	1	1	1	1	H	*Allium*	Grassland	Native
*Ajuga reptans* L.	1	0	0	0	H	*Allium*	Forest	Native
*Alchemilla micans* Buser	1	0	0	1	H	*Allium*	Meadow	Native
*Alliaria petiolata* (M. Bieb.) Cavara& Grande	1	1	1	1	H	*Allium*	Ruderal	Native
*Alnus glutinosa* (L.) Gaertn.	0	1	0	0	PH	*Epilobium*	Forest	Native
*Anemone nemorosa* L.	1	1	0	1	G	*Allium*	Forest	Native
*Arctium tomentosum* Mill.	1	0	0	0	H	*Bidens*	Ruderal	Native
*Arenaria serpyllifolia* L.	0	0	1	1	T	*Allium*	Grassland	Native
*Arrhenatherum elatius* (L.) P. Beauv. ex J. Presl& C. Presl	0	0	1	1	H	*Allium*	Meadow	Native
*Artemisia vulgaris* L.	0	0	0	1	H	*Allium*	Ruderal	Native
*Asarum europaeum* L.	1	1	0	0	H	*Allium*	Forest	Native
*Asperula odorata* L.	1	1	0	0	H	*Bidens*	Forest	Native
*Atriplex patula* L.	0	1	1	1	T	*Allium*	Ruderal	Native
*Avenula pubescens* (Huds.) Dumort.	0	0	1	1	H	*Allium*	Meadow	Native
*Bellis perennis* L.	0	0	1	1	H	*Allium*	Meadow	Native
*Berteroa incana* (L.) DC.	0	0	1	0	T	*Allium*	Ruderal	?
*Bidens frondosa* L.	1	0	0	0	T	*Bidens*	Ruderal	Alien *
*Bromus hordeaceus* L.	0	0	1	0	T	*Allium*	Meadow	Native
*Bromus sterilis* L.	0	0	0	1	T	*Allium*	Ruderal	Alien
*Calystegia sepium* (L.) R. Br.	0	0	1	0	G	*Allium*	Ruderal	Native
*Capsella bursa-pastoris* (L.) Med.	0	0	1	1	T	*Allium*	Ruderal	Alien
*Carex brizoides* L.	1	1	0	0	G	*Allium*	Forest	Native
*Carex hirta* L.	0	0	1	1	G	Allium	Meadow	Native
*Carex ovalis* Good.	1	1	0	1	H	*Allium*	Meadow	Native
*Carex remota* L.	1	1	0	0	H	*Sparganium*	Forest	Native
*Carex* sp.	1	1	1	1	nd	nd	nd	nd
*Carex spicata* Huds.	0	0	1	1	H	*Allium*	Grassland	Native
*Carex sylvatica* Huds.	1	0	0	0	H	*Allium*	Forest	Native
*Carpinus betulus* L.	1	1	1	1	PH	*Epilobium*	Forest	Native
*Cerastium arvense* L.	0	0	1	1	CH	*Allium*	Ruderal	Native
*Cerastium holosteoides* Fr.	0	0	1	1	CH	*Allium*	Meadow	Native
*Cerasus avium* (L.) Moench	1	1	0	0	PH	*Cornus*	Forest	Native
*Chaerophyllum aromaticum* L.	1	0	1	1	H	*Allium*	Ruderal	Native
*Chaerophyllum temulum* L.	0	0	1	0	H	*Allium*	Ruderal	Native
*Chelidonium majus* L.	1	1	0	1	H	*Allium*	Ruderal	Native
*Circaea lutetiana* L.	1	1	0	0	G	*Bidens*	Forest	Native
*Cirsium vulgare* (Savi) Ten.	0	0	1	1	H	*Epilobium*	Ruderal	?
*Convallaria majalis* L.	1	1	0	0	G	*Cornus*	Forest	Native
*Convolvulus arvensis* L.	0	0	1	1	G	*Allium*	Ruderal	Native
*Conyza canadensis*(L.) Cronquist	0	0	1	0	T	*Epilobium*	Ruderal	Alien *
*Cornus sanguinea* L.	1	1	1	1	PH	*Cornus*	Grassland	Native
*Coronilla varia* L.	0	0	1	1	H	*Allium*	Grassland	Native
*Corylus avellana* L.	1	0	0	0	PH	*Cornus*	Forest	Native
*Crataegus* sp.	1	1	1	1	nd	nd	nd	nd
*Crepis biennis* L.	0	0	1	1	H	*Epilobium*	Meadow	Native
*Dactylis glomerata* L.	1	0	1	1	nd	*Allium*	Meadow	Native
*Deschampsia cespitosa* (L.) P. Beauv.	1	1	0	1	H	*Allium*	Meadow	Native
*Dryopteris* sp.	0	1	0	0	nd	nd	nd	nd
*Dryopteris carthusiana* (Vill.) H. P. Fuchs	0	1	0	0	H	*Lycopodium*	Forest	Native
*Duchesnea indica* (Andrews) Focke	0	0	1	1	H	*Cornus*	Ruderal	Alien
*Echium vulgare* L.	0	0	1	0	H	*Allium*	Ruderal	Native
*Elymus repens* (L.) Gould	1	0	1	1	G	*Allium*	Ruderal	Native
*Epilobium montanum* L.	0	0	1	0	H	*Epilobium*	Ruderal	Native
*Equisetum arvense* L.	1	0	0	0	G	*Lycopodium*	Ruderal	Native
*Erigeron annuus* (L.) Pers.	0	0	0	1	H	*Epilobium*	Ruderal	Alien *
*Euonymus europaeus* L.	1	1	0	1	PH	*Cornus*	Forest	Native
*Euonymus verrucosus* Scop.	1	1	0	0	PH	*Cornus*	Forest	Native
*Euphorbia cyparissias* L.	0	0	1	0	G	*Allium*	Grassland	Native
*Euphorbia peplus* L.	0	0	0	1	T	*Allium*	Ruderal	Alien
*Fagus sylvatica* L.	1	1	0	0	PH	*Cornus*	Forest	Native
*Fallopia convolvulus* (L.) Å. Löve	0	0	0	1	T	*Allium*	Ruderal	Alien
*Festuca arundinacea* Schreb.	0	0	1	1	H	*Allium*	Meadow	Native
*Festuca gigantea* (L.) Vill.	1	0	0	1	H	*Allium*	Forest	Native
*Festuca rubra* L.	1	0	1	1	H	*Allium*	Meadow	Native
*Filipendula ulmaria* (L.) Maxim.	1	1	0	0	H	*Allium*	Meadow	Native
*Fragaria viridis* (Duchesne) Weston	1	0	1	1	H	*Cornus*	Grassland	Native
*Fraxinus excelsior* L.	1	1	1	1	PH	*Epilobium*	Forest	Native
*Galeobdolon luteum* Huds.	1	1	0	0	CH	*Allium*	Forest	Native
*Galeopsis tetrahit* L.	1	1	0	0	T	*Allium*	Ruderal	Native
*Galinsoga ciliata* (Raf.) S. F. Blake	0	0	0	1	T	*Epilobium*	Ruderal	Alien *
*Galium aparine* L.	0	1	0	1	T	*Bidens*	Ruderal	Native
*Galium mollugo* L.	0	0	1	1	H	*Allium*	Meadow	Native
*Galium odoratum* (L.) Scop.	1	1	0	0	H	*Bidens*	Forest	Native
*Geranium phaeum* L.	1	1	0	0	H	*Allium*	Forest	Native
*Geranium pratense* L.	0	0	1	1	H	*Allium*	Meadow	Native
*Geranium pusillum* Burm. F. ex L	0	0	1	0	T	*Allium*	Ruderal	Alien
*Geranium robertianum* L.	1	1	0	1	T	*Allium*	Ruderal	Native
*Geum urbanum* L.	1	1	1	1	H	*Bidens*	Ruderal	Native
*Glechoma hederacea* L.	1	1	1	1	H	*Allium*	Ruderal	Native
*Hedera helix* L.	1	1	1	1	PH	*Cornus*	Forest	Native
*Heracleum sphondylium* L.	0	0	0	1	H	*Allium*	Meadow	Native
*Hieracium pilosella* L.	0	0	0	1	H	*Epilobium*	Grassland	Native
*Holcus lanatus* L.	0	0	0	1	H	*Allium*	Meadow	Native
*Humulus lupulus* L.	0	1	0	1	H	*Allium*	Forest	Native
*Impatiens glandulifera* Royle	0	1	0	0	T	*Allium*	Ruderal	Alien *
*Impatiens parviflora* DC.	1	1	0	1	T	*Allium*	Ruderal	Alien *
*Juglans regia* L.	1	0	0	0	PH	*Cornus*	Ruderal	Alien *
*Juncus bufonius* L.	0	0	1	0	T	*Sparganium*	Ruderal	Native
*Juncus tenuis* Willd.	0	0	1	0	H	*Allium*	Meadow	Alien *
*Lactuca serriola* L.	0	0	1	1	H	*Epilobium*	Ruderal	Alien
*Lamium album* L.	0	0	1	1	H	*Allium*	Ruderal	Alien
*Lamium purpureum* L.	0	0	1	0	T	*Allium*	Ruderal	Alien
*Lapsana communis* L.	0	0	1	0	T	*Allium*	Ruderal	Native
*Lathyrus vernus* (L.) Bernh.	1	0	0	0	G	*Allium*	Forest	Native
*Leontodon autumnalis* L.	0	0	1	0	H	*Epilobium*	Meadow	Native
*Leontodon hispidus* L.	0	0	0	1	H	*Epilobium*	Meadow	Native
*Ligustrum vulgare* L.	0	1	0	0	PH	*Cornus*	Grassland	?
*Lolium perenne* L.	0	0	1	1	H	*Allium*	Meadow	Native
*Lonicera xylosteum* L.	1	0	0	0	PH	*Cornus*	Forest	Native
*Lysimachia nummularia* L.	1	1	1	1	H	*Allium*	Meadow	Native
*Lysimachia vulgaris* L.	1	0	0	1	G	*Allium*	Meadow	Native
*Maianthemum bifolium* (L.) F. W. Schmidt	1	1	0	0	G	*Cornus*	Forest	Native
*Medicago lupulina* L.	0	0	1	1	T	*Allium*	Ruderal	Native
*Medicago falcata* L	0	0	1	1	H	*Allium*	Grassland	Native
*Melandrium album* (Mill.) Garcke	0	0	1	0	H	*Allium*	Ruderal	Alien
*Melica nutans* L.	1	0	0	0	H	*Allium*	Forest	Native
*Mentha* sp.	0	0	1	0	nd	nd	nd	nd
*Mercurialis perennis* L.	1	1	0	0	G	*Allium*	Forest	Native
*Milium effusum* L.	1	1	0	0	H	*Allium*	Forest	Native
*Moehringia trinervia* (L.) Clairv.	0	1	0	0	H	*Allium*	Ruderal	Native
*Myosotis arvensis* (L.) Hill	0	0	0	1	T	*Allium*	Ruderal	Alien
*Oxalis acetosella* L.	1	1	0	0	G	*Allium*	Forest	Native
*Oxalis fontana* Bunge	0	0	1	1	G	*Allium*	Ruderal	Alien *
*Padus avium* Mill.	1	1	0	0	PH	*Cornus*	Forest	Native
*Padus serotina* (Ehrh.) Borkh.	1	1	0	1	PH	*Cornus*	Forest	Alien *
*Paris quadrifolia* L.	1	0	0	0	G	*Cornus*	Forest	Native
*Plantago lanceolata* L.	0	0	1	1	H	*Allium*	Meadow	Native
*Plantago major* L.	1	0	1	1	H	*Allium*	Meadow	Native
*Poa annua* L.	1	1	1	1	H	*Allium*	Meadow	Native
*Poa nemoralis* L.	1	0	0	0	H	*Allium*	Forest	Native
*Poa pratensis* L.	0	1	1	1	H	*Allium*	Meadow	Native
*Poa trivialis* L.	1	1	1	1	H	Allium	Meadow	Native
*Polygonatum multiflorum* (L.) All.	1	1	0	0	G	*Cornus*	Meadow	Native
*Polygonatum odoratum* (Mill.) Druce	1	0	0	0	G	*Cornus*	Grassland	Native
*Polygonum aviculare* L.	0	0	1	1	T	*Allium*	Ruderal	Native
*Polygonum lapathifolium* L.	0	0	1	1	T	*Sparganium*	Ruderal	Native
*Potentilla anserina* L.	0	0	1	0	H	*Allium*	Meadow	Native
*Potentilla arenaria* P. Gaertn., B. Mey. &Scherb	0	0	1	1	H	*Allium*	Grassland	Native
*Potentilla argentea* L.	0	0	1	0	H	*Allium*	Grassland	Native
*Potentilla reptans* L.	0	0	1	1	H	*Allium*	Meadow	Native
*Prunella vulgaris* L.	1	0	1	1	H	*Allium*	Meadow	Native
*Prunus domestica* L.	0	1	0	1	PH	*Cornus*	Ruderal	Alien
*Pteridium aquilinum* (L.) Kuhn	0	1	0	0	G	*Lycopodium*	Forest	Native
*Pulmonaria obscura* Dumort.	1	1	0	0	H	*Allium*	Forest	Native
*Quercus robur* L.	1	1	1	0	PH	*Cornus*	Forest	Native
*Quercus rubra* L.	1	1	1	1	PH	*Cornus*	Forest	Alien *
*Quercus petraea* (Matt.) Liebl.	0	1	0	0	PH	*Cornus*	Forest	Native
*Ranunculus acris* L.	1	0	0	1	H	*Allium*	Meadow	Native
*Ranunculus lanuginosus* L.	0	1	0	1	H	*Allium*	Forest	Native
*Ranunculus repens* L.	0	1	1	1	H	*Allium*	Meadow	Native
*Ribes uva-crispa* L.	1	1	0	0	PH	*Cornus*	Forest	Native
*Robinia pseudoacacia* L.	0	0	1	1	PH	*Allium*	Forest	Alien *
*Rubus caesius* L.	1	1	1	1	PH	*Cornus*	Ruderal	Native
*Rubus idaeus* L.	1	1	0	0	PH	*Cornus*	Ruderal	Native
*Rubus* sp.	1	1	0	1	nd	nd	nd	nd
*Rumex obtusifolius* L.	0	0	1	1	H	*Allium*	Ruderal	Native
*Rumex thyrsiflorus* Fingerh.	0	0	1	0	H	*Allium*	Meadow	?
*Sagina procumbens* L.	0	0	1	0	CH	*Allium*	Meadow	Native
*Sambucus nigra* L.	1	1	0	1	PH	*Cornus*	Ruderal	Native
*Setaria viridis* (L.) P. Beauv.	0	0	1	0	T	*Bidens*	Ruderal	Alien *
*Silene nutans* L.	0	0	0	1	H	*Allium*	Grassland	Native
*Solidago canadensis* L.	0	0	1	0	H	*Epilobium*	Ruderal	Alien *
*Solidago gigantea* Aiton	1	0	0	1	H	*Epilobium*	Ruderal	Alien *
*Sorbus aucuparia* L.	1	0	0	0	PH	*Cornus*	Forest	Native
*Stachys sylvatica* L.	0	1	0	0	H	*Allium*	Forest	Native
*Stellaria graminea* L.	0	0	1	0	H	*Allium*	Meadow	Native
*Stellaria holostea* L.	1	0	0	1	CH	*Allium*	Forest	Native
*Stellaria media* (L.) Vill.	1	1	1	1	T	*Allium*	Ruderal	Native
*Symphytum tuberosum* L.	1	1	0	0	H	*Allium*	Forest	Native
*Taraxacum officinale* F.H. Wiggers	1	0	1	1	H	*Epilobium*	Meadow	Native
*Thymus serpyllum* L.	0	0	1	1	CH	*Allium*	Grassland	Native
*Tilia cordata* Mill.	0	1	0	1	PH	*Epilobium*	Forest	Native
*Tilia* sp.	1	1	0	0	PH	nd	nd	nd
*Trifolium pratense* L.	0	0	1	1	H	*Allium*	Meadow	Native
*Trifolium repens* L.	0	0	1	1	H	*Allium*	Meadow	Native
*Tussilago farfara* L.	1	0	0	0	H	*Epilobium*	Ruderal	Native
*Ulmus laevis* Pall.	0	0	1	1	PH	*Epilobium*	Forest	Native
*Ulmus minor* Mill.	1	0	0	0	PH	*Epilobium*	Forest	Native
*Urtica dioica* L.	1	1	1	1	H	*Allium*	Ruderal	Native
*Veronica arvensis* L.	0	0	1	1	T	*Allium*	Ruderal	Alien
*Veronica chamaedrys* L	1	0	1	1	H	*Allium*	Grassland	Native
*Veronica serpyllifolia* L.	0	0	1	1	H	*Allium*	Ruderal	Native
*Viciatetrasperma* (L.) Schreb.	0	0	0	1	T	*Allium*	Ruderal	Alien
*Viola odorata* L.	1	0	1	1	H	*Allium*	Ruderal	?
*Viola reichenbachiana* Jord. ex Boreau	1	1	0	1	H	*Allium*	Forest	Native
*Viola* sp.	0	0	1	0	nd	nd	nd	nd
